# Muscle strength and areal bone mineral density at the hip in women: a cross-sectional study

**DOI:** 10.1186/s12891-015-0586-2

**Published:** 2015-05-24

**Authors:** Julie A. Pasco, Kara L. Holloway, Sharon L. Brennan-Olsen, David J. Moloney, Mark A. Kotowicz

**Affiliations:** Epi-Centre for Healthy Ageing, IMPACT SRC, School of Medicine, Deakin University, PO Box 281, Geelong, VIC 3220 Australia; Department of Medicine, NorthWest Academic Centre, The University of Melbourne, St Albans, VIC Australia; University Hospital Geelong, Barwon Health, Geelong, VIC Australia

**Keywords:** Body composition, Bone mineral density, Lean mass, Muscle strength, Women

## Abstract

**Background:**

Muscle strengthening exercises are promoted for building and maintaining a healthy skeleton. We aimed to investigate the relationship between muscle strength and areal bone mineral density (BMD) at the hip in women aged 26–97 years.

**Methods:**

This cross-sectional study utilises data from 863 women assessed for the Geelong Osteoporosis Study. Measures of hip flexor and abductor strength were made using a hand-held dynamometer (Nicholas Manual Muscle Tester). The maximal measure from three trials on each leg was used for analyses. BMD was measured at the hip using dual energy x-ray absorptiometry (DXA; Lunar DPX-L). Total lean mass, body fat mass and appendicular lean mass were determined from whole body DXA scans. Linear regression techniques were used with muscle strength as the independent variable and BMD as the dependent variable. Models were adjusted for age and indices of body composition.

**Results:**

Measures of age-adjusted hip flexor strength and hip abductor strength were positively associated with total hip BMD. For each standard deviation (SD) increase in hip flexor strength, the increase in mean total hip BMD (SD) was 10.4 % (p = 0.009). A similar pattern was observed for hip abductor strength, with an increase in mean total hip BMD of 22.8 % (p = 0.025). All associations between hip muscle strength and total hip BMD were independent of height, but were nullified after adjusting for appendicular lean mass or total lean mass.

**Conclusions:**

There was a positive association observed between muscle strength and BMD at the hip. However, this association was explained by measures of lean mass.

## Background

Recent recommended guidelines in Australia for building and maintaining healthy bones include regular weight bearing activities and muscle strengthening exercises [1]. Skeletal muscle strength is a measure of how much force the muscle can exert and is reliant on fast twitch fibres which respond to short bursts of energy. Exercises that exert extraordinary mechanical strains on bone and apply high intensity loading are required to elicit a response [2]. Contracting skeletal muscle also produces hormonal and nervous stimuli that contribute to the muscle-bone interaction [3, 4].

Meta-analyses reveal that a combination of high impact and high-magnitude loading exercises are most effective in enhancing areal bone mineral density (BMD) at the hip and spine in premenopausal women [5] and for reducing bone loss at these sites in postmenopausal women [6]. Such reviews provide evidence that regular weight bearing activities and muscle strengthening exercises have beneficial skeletal effects but what remains uncertain is whether it is an improvement in muscle strength or muscle mass that impacts on bone. As a first step in exploring the muscle-bone relationship, we aimed to investigate the cross-sectional associations between hip flexor and hip abductor muscle strength and BMD at the total hip in women aged 26–97 years.

## Methods

### Subjects

This cross-sectional study is set in the Barwon Statistical Division in south-eastern Australia and utilises data from the 6-year follow-up phase of the Geelong Osteoporosis Study (GOS). An age-stratified sample of 1494 women was selected at random from Commonwealth electoral rolls and recruited into the GOS, with a participation of 77.1 % during the years 1993–1997 [7]. In Australia, registration with the Australian Electoral Commission is compulsory for adults aged 18 years and over, so the electoral roll provides a comprehensive listing of all residents. From 1217 women who were available for the 6-year follow-up phase of the study, 1051 women participated commencing in the year 2000. Most (99 %) of the cohort was white; details of recruitment and retention have been published elsewhere [7]. For this study, 188 women were excluded because of incomplete data concerning dual energy x-ray absorptiometry (DXA) and/or muscle strength. Thus, 863 women, aged 26–97 years, were eligible for this analysis.

The study was approved by the Barwon Health Human Research Ethics Committee. All study participants provided informed, written consent.

### Measures

Measures of maximal isometric strength of the hip flexors and hip abductors on both legs were determined using a hand-held dynamometer (Nicholas Manual Muscle Tester, MMT; Lafayette Instruments, Lafayette, IN). The MMT registers the break force (kg) applied by the tester to oppose the subject's efforts to sustain the position of a raised limb [8]. The testing procedure was explained and demonstrated to the subjects before the trials. Muscle groups were assessed in separate trials in triplicate on each leg. The maximum recorded value for each muscle group was used in analyses. Multiplying the maximal registered force (kg) by 9.81 converted the force to newtons (N). There were missing muscle strength measures for hip flexors (n = 1) and hip abductors (n = 7).

Height and weight were measured to the nearest ± 0.001 m and ± 0.1 kg, respectively. DXA (Lunar DPX-L, Madison, WI, USA) provided measures of areal BMD at the proximal femur (femoral neck, Ward’s triangle and trochanter); total hip BMD was calculated from the sub-regions [9]. BMD values for osteoporosis at the total hip corresponded to T-scores < −2.5 according to the Australian reference ranges for women [10]. Whole body scans provided measures of lean tissue and body fat mass. Lean tissue assessed by DXA technology comprises non-fat and non-bone tissue and correlates well with skeletal muscle mass measured using magnetic resonance imaging [11]. Appendicular lean mass (ALM, in kg) was determined by summing lean mass measures for the arms and legs. Low ALM was recognised for T-scores < −2.0 according to published reference ranges [12].

### Statistical analysis

For descriptive purposes, high muscle strength was assigned for subjects with values in the upper tertile of the distribution for hip flexor or hip abductor strength; mid and low muscle strength referred to measures in the mid and lower tertiles, respectively. Differences in subject characteristics according to muscle strength categories were identified using analysis of variance (ANOVA) for parametric or the Kruskal-Wallis test for non-parametric continuous data. Pearson’s correlation coefficients were determined for analysis of associations between age, anthropometry, indices of body composition and muscle strength. Associations between muscle strength (or mass) and BMD were determined using linear regression techniques; in statistical models, muscle strength (or mass) was the independent variable and BMD the dependent variable. Body composition variables were expressed in standard deviation (SD) units. Higher than linear adjustments made for age were centred about the mean to reduce collinearity. Interaction terms were tested in the models to identify effect modifiers. All statistical analyses were performed using Minitab (version 16; Minitab, State College, PA, United States of America).

## Results

Subject characteristics are shown in Table 1. Hip flexor strength was negatively correlated with age (r = −0.49, p < 0.001), positively with appendicular lean mass (r = +0.39, p < 0.001), total lean mass (r = +0.35, p < 0.001), body fat mass (r = +0.05, p = 0.132), hip abductor strength (r = +0.43, p < 0.001) and total hip BMD (r = +0.32, p < 0.001). Similarly, hip abductor strength was negatively correlated with age (r = −0.41) and positively with appendicular lean mass (r = +0.36), total lean mass (r = +0.33), body fat mass (r = +0.11, p = 0.001) and total hip BMD (r = +0.29, p < 0.001). Compared to women with high muscle strength (upper tertile), those with decreasing muscle strength (mid and low tertiles) were progressively older, shorter, weighed less and had lower appendicular lean mass, total lean mass and total hip BMD.

**Table 1:** Subject characteristics

	All	Hip flexor strength*				Hip abductor strength*			
		High	Mid	Low	p-value	High	Mid	Low	p-value
	n = 863	n = 287	n = 287	n = 288		n = 285	n = 286	n = 285	
Age (yr)	57 (44–70)	47 (38–59)	55 (44–67)	70 (57–78)	<0.001	49 (42–60)	56 (44–68)	69 (55–78)	<0.001
Weight (kg)	70.4 (±13.9)	72.8 (±14.3)	70.7 (±13.4)	67.8 (±13.5)	<0.001	73.1 (±14.3)	71.0 (±14.0)	67.0 (±12.5)	<0.001
Height (m)	1.60 (±0.07)	1.62 (±0.06)	1.60 (±0.06)	1.59 (±0.07)	<0.001	1.62 (±0.06)	1.61 (±0.06)	1.58 (±0.07)	<0.001
Body mass index (kg/m^2^)	27.4 (±5.3)	27.8 (±5.2)	27.6 (±5.3)	26.9 (±5.2)	0.137	27.9 (±5.4)	27.5 (±5.5)	26.8 (±4.9)	0.051
Appendicular lean mass (kg)	17.4 (±2.4)	18.5 (±2.3)	17.4 (±2.1)	16.6 (±2.3)	<0.001	18.3 (±2.3)	17.5 (±2.3)	16.3 (±2.2)	<0.001
Total lean mass (kg)	38.7 (±4.3)	40.4 (±4.2)	38.7 (±3.8)	37.1 (±4.2)	<0.001	40.2 (±4.0)	38.9 (±4.1)	37.1 (±4.1)	<0.001
Body fat mass (kg)	28.3 (±10.5)	28.8 (±10.7)	28.6 (±10.4)	27.6 (±10.4)	0.324	29.6 (±10.9)	28.7 (±10.6)	26.5 (±9.6)	0.001
Total hip BMD (g/cm^2^)	0.966 (±0.163)	1.020 (±0.142)	0.975 (±0.161)	0.903 (±0.164)	<0.001	1.019 (±0.143)	0.962 (±0.160)	0.916 (±0.168)	<0.001
Hip flexor strength (N)	163 (±53)	221 (±32)	161 (±13)	106 (±24)	<0.001	184 (±48)	169 (±49)	133 (±49)	<0.001
Hip abductor strength (N)	134 (±51)	154 (±51)	142 (±48)	108 (±42)	<0.001	192 (±38)	127 (±12)	84 (±20)	<0.001

*missing data: hip flexor strength (n = 1), hip abductor strength (n = 7)

Characteristics are shown for the whole group and for those with high (upper tertile) vs mid (mid tertile) and low (low tertile) measures for hip flexor and hip abductor strength. The data are presented as mean (±SD) or median (interquartile range)

**Fig. 1:**
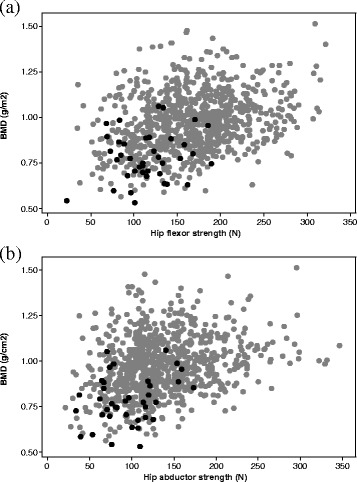
The relationship of total hip BMD with (**a**) hip flexor strength and (**b**) hip abductor strength. Black symbols represent individuals with low appendicular lean mass (T-score < −2 according to published reference ranges [12]); data for all other individuals are shown in grey

Forty-six (5.3 %) women had osteoporosis and, among those, only one had high hip flexor strength and two had high hip abductor strength. Similarly, 39 (4.5 %) women had low appendicular lean mass and, among those, two had high hip flexor strength and four had high hip abductor strength. The positive relationship between muscle strength and total hip BMD is presented in Fig. 1, which also shows that individuals with low appendicular mass (black symbols) clustered in the lower ranges for both muscle strength and BMD.

After adjusting for age, hip flexor strength remained positively associated with total hip BMD. For each SD increase in hip flexor strength there was an increase in mean total hip BMD (SD) of 10.4 % (Table 2). The association was independent of height and body fat mass, but the association was lost after further adjustment for appendicular lean mass or total lean mass. Similarly, hip abductor strength was positively associated with age-adjusted BMD. For each SD increase in hip abductor strength, there was an increase in mean BMD (SD) of 22.8 %. The associations were independent of height, but attenuated after adjusting for body fat mass (p = 0.093) and lost after adjustment for appendicular lean mass or total lean mass. No interactions were detected between age and muscle strength in the association with total hip BMD.

**Table 2:** Results of linear regression models showing the relationship between muscle strength and bone mineral density

Dependent variable	Hip flexors			Hip abductors		
	β coefficient	se (β)	*p* value	β coefficient	se (β)	*p* value
Total hip BMD	0.104	0.040	0.009	0.228	0.102	0.025

Models are adjusted for age. Bone mineral density (BMD) and muscle strength are expressed in standard deviation units. Models were adjusted for AgeC and (AgeC)^2^ where age was centred about the mean (57 years)

Appendicular lean mass was positively associated with total hip BMD before (r = +0.51) and after adjusting for age; each SD increase in appendicular lean mass was associated with a mean BMD (SD) increase of 44.8 % (age-adjusted β coefficient 0.448, se (β) 0.037, p < 0.001). Similarly, there was a positive association between total lean mass and total hip BMD (SD) (age-adjusted β coefficient 0.417, se (β) 0.038, p < 0.001). These associations were independent of height and no interactions were detected between age and lean mass in the associations with total hip BMD.

## Discussion

In this cross-sectional study, we demonstrate a positive association between muscle strength and BMD. However, the association was explained by differences in appendicular or total lean mass. The hip plays an important role in raising the leg towards the torso and for keeping hips and lower back properly aligned during movement. As the hip flexor muscle group connects the femur with the pelvis and lumbar spine, weakness of this group of muscles leads to poor balance and postural problems that can cause difficulties when standing or walking for extensive periods of time. The hip abductors move the leg out to the side and thus play an important role in stabilising the tilt of the pelvis in the frontal plane. Weakness in these muscles limits the stride and alters gait, and causes an abnormal walking pattern known as the Trendelenburg gait [13]. As well as modifying gait, these muscle groups can impact on the skeleton through loading forces and by expressing growth factors which have an anabolic effect on bone.

A program of high impact vertical jumping exercises has been shown to increase BMD at the proximal femur for premenopausal, but not postmenopausal, women [14]. However, high-intensity strength training has been demonstrated to increase muscle mass, muscle strength and BMD at the lumbar spine and femoral neck among postmenopausal women [15]. It remains unclear, however, whether the forces applied to the skeleton during such exercises are related to muscle strength or muscle mass.

A Turkish study of sedentary postmenopausal women measured hip and trunk muscle as well as grip strength to explore associations with BMD at the lumbar spine, proximal femur and distal radius [16]. Only one combination of measurements was significant, namely, hip abductor strength was positively associated with BMD at the femoral neck. A more recent study that involved men aged 40–79 years, from the United Kingdom and Belgium, reported that relative appendicular muscle mass (appendicular lean mass/height^2^) and fat mass were both positively associated with BMD [17]. In a further study that investigated the effect of leg muscle mass, knee extensor length and fat infiltration of muscle (measured by computed tomography) on femoral neck BMD in Korean men and women aged over 65 years, all three variables were positively associated with BMD in both sexes [18]. In accordance with our study, they found that after adjustment for muscle mass, muscle strength became a non-significant predictor of BMD.

A large Finnish study of postmenopausal women aged 63–75 years measured muscle strength, lean tissue distribution and overall body composition to determine if these variables could be used as indicators of osteoporosis [19]. The authors reported that, compared to women without osteoporosis, those with osteoporosis had lower lean mass index, appendicular muscle mass, grip strength and knee extension strength, but no difference in fat mass index was observed. Grip strength and knee extension strength were 19 % and 16 % lower, respectively, in women with osteoporosis compared to those without. The authors also considered a multivariate model, which adjusted for age, grip strength, leg extension strength, fat mass index, lean mass index, number of medications, alcohol consumption, current smoking, dietary calcium intake and hormone therapy. After these adjustments, only grip strength, leg extension length and years of hormone therapy remained significant indicators of osteoporosis. In contrast to our study, the authors concluded that muscle strength and lean mass were independently associated with BMD. They also considered that for these older women, muscle mass and strength were more important than body weight for maintaining BMD in the normal (healthy) range.

Our findings suggest that the association observed between muscle strength and BMD at the hip might be driven by associated differences in the quantity of muscle. There is some evidence to support this notion. The anabolic steroid, nandrolone, has positive effects on muscle growth and clinical trials suggest it might be a useful agent for treating osteoporosis. Among elderly women with osteoporosis, injections of nandrolone decanoate were associated with measurable gains in BMD at the lumbar spine and femoral neck [20, 21]. These studies also reported increases in muscle mass after treatment but it remains unclear whether there were associated increases in muscle strength.

The main strength of our study is that participants were drawn from the general population and spanned the full adult age range. In our study, age was not identified as an effect modifier, suggesting that the relationships between muscle strength, lean mass and total hip BMD were consistent across the age range. Nevertheless, as this is a cross-sectional analysis of data collected at a follow-up phase of a cohort study, we cannot exclude the possibility that there may have been differential loss to follow-up related to these musculoskeletal parameters. Another strength of the study is that muscle strength, muscle mass and BMD were measured at the same, clinically relevant site. However, we acknowledge that measurement of hip extension muscle strength might have enhanced the data for addressing the study aims. Further studies are required that measure muscle mass and strength in the same muscle groups together with BMD assessment at the most appropriate skeletal sites. As the data were collected from an essentially white sample of Australian women, our findings might not be generalizable to different female populations nor to men. Also, as we did not measure osteogenic factors, such as insulin-like growth factor-1, which are secreted by skeletal muscle [3, 4], further studies should explore their contribution to the muscle-bone interaction during exercise.

## Conclusions

Within these constraints, however, we report that the observed association between muscle strength, for hip flexors and abductors, and total hip BMD in women was nullified after adjusting for lean mass. Therefore, it seems that lean mass, rather than muscle strength, impacts on bone density.

## Abbreviations

ALM: Appendicular lean mass
ANOVA: Analysis of variance
BMD: Bone mineral density
DXA: Dual energy x-ray absorptiometry
GOS: Geelong osteoporosis study
MMT: Manual muscle tester
SD: Standard deviation
